# Diaphragmatic and Pulmonary Functions Following an Ultrasound-Guided Supraclavicular Approach Versus a Costoclavicular Approach of a Brachial Plexus Block: A Randomized Study

**DOI:** 10.7759/cureus.62586

**Published:** 2024-06-18

**Authors:** Rajkumar K Saraswat, Mangilal Deganwa, Kalpana Verma, Avnish Bharadwaj

**Affiliations:** 1 Anaesthesiology and Critical Care, Mahatma Gandhi Medical College and Research Institute, Jaipur, IND

**Keywords:** supraclavicular block, costoclavicular block, below shoulder surgery, regional anaesthesia, pulmonary function, diaphragm, brachial plexus block

## Abstract

Introduction: A costoclavicular brachial plexus block is an emerging infraclavicular approach that targets the cords lateral to the axillary artery, providing rapid onset of sensory-motor blockade. However, the incidence of hemi-diaphragmatic paralysis (HDP), a potential complication, remains unclear compared to the widely used supraclavicular (SC) approach. This study aimed to compare the incidence of HDP between ultrasound-guided costoclavicular and SC brachial plexus blocks.

Objectives: To compare the influence of ultrasound-guided SC and costoclavicular brachial plexus blocks on diaphragmatic excursion, thickness, and contractility along with pulmonary function.

Materials and methods: This prospective, randomized, observer-blinded controlled trial included 60 patients undergoing below-shoulder surgeries. Patients were randomized to receive either ultrasound-guided SC (Group S) or costoclavicular (Group C) brachial plexus block with 0.5% levobupivacaine. The diaphragmatic function was assessed using ultrasonographic evaluation of diaphragm thickness and diaphragmatic thickness fraction (DTF) pre- and postblock. Pulmonary function tests (PFTs) (forced vital capacity (FVC), forced expiratory volume in one second (FEV1), and peak expiratory flow rate (PEFR)) were performed preblock and two hours postblock. Block characteristics were compared.

Results: The SC group exhibited a significantly larger reduction in DTF from preblock to postblock compared to the costoclavicular group (mean ΔDTF: 34.38% vs. 14.01%, p<0.01). Both groups showed significant declines in FVC, FEV1, and PEFR postblock, but the magnitude of deterioration was significantly greater in the SC group, displaying no significant difference in block characteristics.

Conclusion: The costoclavicular brachial plexus block demonstrated superior preservation of diaphragmatic contractility and lesser deterioration of PFTs compared to the SC approach while being equally effective. These findings highlight the potential benefits of the costoclavicular technique in minimizing diaphragmatic dysfunction and respiratory impairment, particularly in patients at risk for respiratory complications.

## Introduction

The supraclavicular (SC) block is one of several techniques used to anesthetize the brachial plexus. The block is performed at the level of the brachial plexus trunks where almost the entire sensory, motor, and sympathetic innervation of the upper extremity are carried in just three nerve structures confined to a very small surface area. Consequently, this technique typically provides a predictable, dense block with rapid onset [1]. The SC block (SCB) provides anesthesia and analgesia to the upper extremity below the shoulder. It is an excellent choice for elbow and hand surgery.

The recently described ultrasound-guided costoclavicular brachial plexus block (CC-BPB) is an alternative to the classic lateral infraclavicular fossa block approach and provides adequate anesthesia and analgesia for upper limb surgery [2-5].

The CC-BPB, pioneered by Li et al. [3] and performed at the costoclavicular space (CCS), targets the center of the three neural cords lateral to the axillary artery and has been reported to produce rapid onset times. Although the two approaches require an injection of local anesthetic (LA) close to the contiguous compartments of the brachial plexus, the relevant sono anatomy for both approaches shares a similar connection between the brachial plexus and artery. For the SC approach, the trunks and divisions of the brachial plexus arrange compactly superolateral to the subclavian artery at the SC fossa. Alternately, at the CCS, all three cords of the brachial plexus are clustered together lateral to the axillary artery.

The anatomic relationship of the three cords has been found to be a relatively constant arrangement, in which the medial cord is located at the innermost level. CC-BPB provides a rapid onset of sensory-motor blockade as it is performed at the CCS and targets the center of the three neural cords lateral to the axillary artery. Successful CC-BPB requires a low volume of LA for surgical anesthesia of no more than 25 mL, and it was reported that ultrasound-guided CC-BPB could provide a faster onset of sensory blockade than IC-BPB. Costoclavicular brachial plexus block varies from SCB as all the three cords of the brachial plexus are clustered in the former and the low dose of LA and single injection provides effective analgesia and anesthesia. In the conventional SC approach, the brachial plexus around the subclavian artery is blocked with a higher risk of ulnar nerve sparing and vessel rupture. There is a low risk of vessel rupture and pleural puncture in the costoclavicular variant of infraclavicular brachial plexus block (ICBPB) as the nerve cords are first approached before the vessel and the pleura when compared with other approaches to ICBPB.

The incidence of HDP is reported up to 70% in the SC approach [6,7]. Even though well tolerated in healthy patients, it can be a critical issue in patients with marginal pulmonary function [8]. The phrenic nerve and brachial plexus are within 2 mm of each other at the cricoid cartilage level, with an additional 3 mm separation for every cm more caudal in the neck [9]. The close distance between the block site and the C3-C5 nerve root or the phrenic nerve is thought to be critical in the occurrence of HDP.

Despite the incidence of HDP being lower after the SC brachial plexus block (SCB) than after the inter scalene brachial plexus block, the incidence of HDP after the SCB has been reported to range from 30%-60%. The incidence of hemi-diaphragmatic paralysis (HDP) after ICBPB is even lower, likely due to the relatively long distance between the phrenic nerve and the block site. We have also reported retrospective data using chest radiography, suggesting a lower incidence of HDP in the costoclavicular approach compared to the SC approach.

However, only a few randomized trials have compared the incidence of HDP after costoclavicular and SCB. As the SC approach is the most widely used approach, we compared the costoclavicular block (CCB) with the SCB.

The primary objective of this study was to investigate the influence of CCB and SCB on the diaphragmatic function, in terms of changes in excursion and thickness of the diaphragm measured by ultrasonography, as well as changes in dynamic pulmonary functions measured by spirometry.

The secondary objectives included studying and comparing the onset and duration of sensory and motor blocks following CCB and SCB, as well as examining the complications encountered with both of these techniques.

## Materials and methods

Study design and setting

This was a prospective, randomized, observer-blinded controlled trial conducted at the Department of Anesthesia, Mahatma Gandhi Medical College, Jaipur, India, from September 2022 to February 2024. The study protocol was approved by the Institutional Ethics Committee, Mahatma Gandhi Medical College and Research Institute, Jaipur, (letter number: 975). This trial was registered on the Clinical Trials Registry, India (CTRI/2023/02/049566).

Sample size

Sixty consenting patients fulfilling criteria undergoing below shoulder surgeries under ultrasound-guided supraclavicular and CC-BPB during the study period. Randomization was done using the computer box method.

Inclusion criteria

The study included patients scheduled for elective upper limb surgeries, including procedures involving the elbow, forearm, hand, and wrist. Both male and female patients were eligible to participate. The age range for participants was between 18 and 65 years with a body mass index (BMI) between 18.5 and 24.9 kg/m^2^. Only those classified as American Society of Anesthesiologists (ASA) physical status I or II were included in the study.

Exclusion criteria

Patients with any bleeding disorders or those currently on anticoagulants, sedatives, or antipsychotics were excluded from the study. Individuals with neurological deficits involving the brachial plexus, as well as those with allergies to local anesthetics or infections at the injection site, were excluded. Additionally, patients who required conversion to general anesthesia, pregnant and lactating women, and individuals with significant pulmonary disease - including all pulmonary morbidities where respiratory compromise was expected in the case of HDP - were excluded from the study.

Procedure

After a thorough pre-anesthetic check-up the day before, patients were transferred to the operating room on the day of surgery. Upon arrival in the preoperative holding area, patients were randomly allocated to either Group C or Group S. Group C (n=30) received an ultrasound-guided CCB with 0.5% levobupivacaine. Group S (n=30) received an ultrasound-guided SCB with 0.5% levobupivacaine.

The "ABCDE" method, as described by Tsui et al. [10], is a structured ultrasonographic approach used to assess diaphragmatic motion and contraction with the Sonosite Turbo M ultrasound device (Fujifilm Holdings Corporation, Tokyo, Japan). This method involves several steps. First, the ultrasound probe is positioned at the zone of apposition of the anterior axillary line. Next, lung sliding is observed, which indicates breathing movement. The probe is then moved caudally to locate the diaphragm for evaluation. During full inspiration, when the subject holds their breath at total lung capacity, the diaphragm's thickening will be visible on the ultrasound, signifying its shortening and contracting action. Finally, the diaphragm thickening fraction (DTF) is calculated based on the change in diaphragm thickness from expiration to inspiration relative to the thickness at expiration, using the formula:

Diaphragm thickness fraction (DTF) = Thickness at inspiration − Thickness at expiration / Thickness at expiration

Pulmonary function tests (PFT) were performed on elective pre-operative and postblock patients in a semi-recumbent position in the procedure area using the RMS Helios 702 portable spirometer. The parameters recorded were forced vital capacity (FVC), forced expiratory volume in one second (FEV1), and peak expiratory flow rate (PEFR). After a thorough pre-anesthetic check-up a day before, patients were shifted to the operating room on the day of surgery, where they were randomly allocated to either group.

Table 1 presents the technique of anesthesia.

**Table 1:** Technique of anesthesia

	Costoclavicular Block	Supraclavicular Block
Patient Positioning	Supine, arm abducted at 90 degrees	Supine, with a soft pad under the back, head turned contralaterally
Ultrasound Guidance Initial Scan	Below the midpoint of the clavicle, over the medial infraclavicular fossa, tilted cephalad	Above the clavicle, tilted caudally towards the first rib
Target	Costoclavicular space between the clavicle and second rib, until all three cords visualized laterally to the axillary artery	Corner pocket (intersection between the first rib and subclavian artery)
Needle Placement	In-plane cranial to caudal, tip in the middle of the three cords	Directed first towards the corner pocket, then towards neural cluster formed by trunks and divisions
Anaesthetic Administration	20 mL of 0.5% levobupivacaine between the cords	20 mL of 0.5% levobupivacaine between trunks or divisions

**Figure 1:**
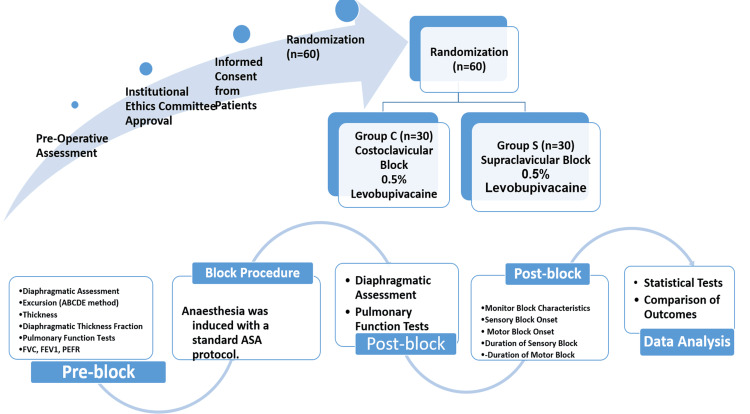
Study vignette ASA: American Society of Anesthesiologists; FVC: Forced vital capacity; FEV1: Forced expiratory volume in 1 second; PEFR: Peak expiratory flow ratio

The block was given with a standard ASA protocol. After administering the block, the progress of the block was monitored using parameters such as sensory block onset, motor block onset, duration of sensory block, and duration of motor block. Diaphragmatic function and pulmonary functions were assessed preoperatively (preblock) and again two hours after administering the block. The primary outcome was to assess the influence of both blocks on diaphragmatic function in terms of changes in excursion and thickness of the diaphragm as measured by ultrasonography. The secondary outcome involved comparing the onset and duration of sensory and motor blocks following both techniques and studying the complications encountered with the two techniques (Figure 1).

Analysis

Data were entered into Microsoft Excel (Microsoft® Corp., Redmond, WA), and statistical tests, including the Mann-Whitney U test, Independent t-test, and Wilcoxon signed-rank tests, were applied based on the type of distribution of data, and results were calculated. A p value < 0.05 was considered as significant at 95% CI.

## Results

The distribution of patients across different age groups was comparable between the two arms. Around 60-63% of patients were aged 21-40 years in both groups. Fisher's exact test showed no statistically significant difference in the age distribution (p=0.56). There was a significant difference in gender distribution between the groups (p<0.05, chi-square test). In Group C, 60% were males, while Group S had a higher proportion of males at 83.3%. The BMI profiles were similar across groups. Most patients had normal BMI (18.5-22.9 kg/m^2^), followed by overweight and obese categories in both arms. No significant difference in BMI distribution according to Fisher's exact test (p=0.59). The majority of patients in both Group C (80%) and Group S (83.3%) belonged to ASA grade I. Only 20% and 18.3% were ASA II in Groups C and S, respectively. The difference was not statistically significant (p=0.73). The two study groups were well-matched for baseline age, BMI, and ASA grade distributions. However, there was a statistically significant difference in gender proportion with a higher percentage of males in the supraclavicular group (Table 2).

**Table 2:** Comparison of baseline details of patients in both groups (n=60)

		Group C (Costoclavicular block) [n=30]		Group S (Supraclavicular block) [n=30]		Test statistics
		n	%	n	%	
Age group (years)	≤ 20	02	6.7	05	16.7	Fisher exact value=2.22, df=3, p value=0.56
	21-40	19	63.3	18	60	
	41-60	06	20	06	20	
	>60	03	10	01	3.3	
Gender	Male	18	60	25	83.3	χ^2 ^=4.02, df=1, p value<0.05
	Female	12	40	05	16.7	
BMI (kg/m^2^)	<18.5	0	0.00	1	3.33	Fisher exact value=2.18, df=3, p value=0.59
	18.5–22.9	13	43.33	16	53.33	
	23–24.9	09	30.00	06	20.00	
	≥25	08	26.67	07	23.33	
ASA Grade	ASA I	24	80	25	83.3	χ^2 ^=0.11, df=1, p value=0.73
	ASA II	06	20	05	18.3	

Table 3 compares the diaphragm thickness during inspiration and expiration, before and after giving the nerve block, between the CCB (Group C) and SCB (Group S) groups. Before the block, the median inspiratory diaphragm thickness was 3.35 mm in Group C and 3.6 mm in Group S. The difference was not statistically significant (p=0.08). The median expiratory diaphragm thickness before block was 1.75 mm and 2 mm in Groups C and S, respectively. The difference did not reach statistical significance (p=0.06).

**Table 3:** Analysis of diaphragm thickness among both groups of patients (n=60)

Time	Diaphragm thickness (mm)								Test statistics Mann Whitney U test	
	Group C (Costoclavicular block) [n=30]				Group S (Supraclavicular block) [n=30]					
	Mean	SD	Median	IQR	Mean	SD	Median	IQR	Z value	p value
Preblock inspiration	3.34	0.54	3.35	0.9	3.58	0.57	3.6	0.7	-1.7	0.08
Preblock expiration	1.78	0.3	1.75	0.4	1.94	0.36	2	0.5	-1.86	0.06
Postblock inspiration	3.11	0.65	3.1	1.2	3.76	1.06	3.6	1	-2.91	<0.01
Postblock expiration	1.79	0.34	1.8	0.6	2.54	0.92	2.3	0.6	-4.46	<0.01
Δ Inspiration	0.22	0.41	0.2	0.3	-0.17	0.95	-0.2	0.65	-2.8	<0.01
Δ Expiration	-0.01	0.26	0	0.23	-0.6	0.76	-0.5	0.55	-4.48	<0.01

After giving the nerve block, the median inspiratory thickness reduced to 3.1 mm in Group C but increased to 3.6 mm in Group S. This difference was statistically highly significant (p<0.01). The median expiratory thickness changed to 1.8 mm in Group C but increased to 2.3 mm in Group S postblock. This difference was also highly significant statistically (p<0.01). The median change (Δ) in inspiratory and expiratory thickness from preblock to postblock showed statistically significant reductions in Group C compared to Group S (p<0.01). Diaphragm thickness was significantly preserved in the costoclavicular group compared to reductions seen in the supraclavicular group after the nerve blocks. This indicates less diaphragmatic dysfunction with the costoclavicular approach (Table 3).

In the costoclavicular group (Group C), the median inspiratory diaphragm thickness reduced significantly from 3.35 mm pre-block to 3.1 mm post-block (p<0.01). The median expiratory thickness in this group changed from 1.75 mm to 1.8 mm post-block. However, this difference was not statistically significant (p=0.2). In the supraclavicular group (Group S), the median inspiratory thickness did not change significantly pre- and postblock (3.6 mm at both times, p=0.67). However, the median expiratory thickness increased significantly from 2 mm pre-block to 2.3 mm post-block.

Table 4 shows that CCB led to a significant reduction in inspiratory diaphragm thickness indicating preserved diaphragmatic function without any paresis or less paresis. Supraclavicular block caused no change in inspiratory thickness indicating phrenic nerve paresis (Table 4).

**Table 4:** Analysis of preblock and postblock diaphragm thickness among both the groups (n=60)

	Respiration Stage	Group	Preblock		Postblock		Test Statistics Wilcoxon Signed-Rank Test	
			Median	IQR	Median	IQR	Z value	p value
Diaphragm thickness (mm)	Inspiration	Group C	3.35	0.9	3.1	1.2	-3.34	<0.01
		Group S	3.6	0.7	3.6	1	-0.41	0.67
	Expiration	Group C	1.75	0.4	1.8	0.6	-1.23	0.2
		Group S	2	0.5	2.3	0.6	-4.09	<0.01
Diaphragmatic thickness fraction (DTF)	-	Group C	86.6	14.5	75	19.9	-4.76	<0.01
		Group S	86.95	17.1	53.2	29.1	-4.78	<0.01

In Group C, the mean preblock DTF was 88.15%, which reduced significantly to 74.14% postblock (p<0.01). In Group S, the mean preblock diaphragmatic thickness fraction (DTF) was 85.64%, which decreased significantly to 51.26% after the block (p<0.01). Though DTF declined significantly in both groups, the decline in Group S (SCB) was much larger compared to Group C (CCB). The median DTF dropped from 86.6% to 75% in Group C, compared to a larger fall from 86.95% to 53.2% in Group S. In summary, diaphragmatic contractility assessed by DTF was better preserved in the costoclavicular group compared to the significant deterioration observed in the supraclavicular group following the nerve blocks (Table 4).

**Figure 2:**
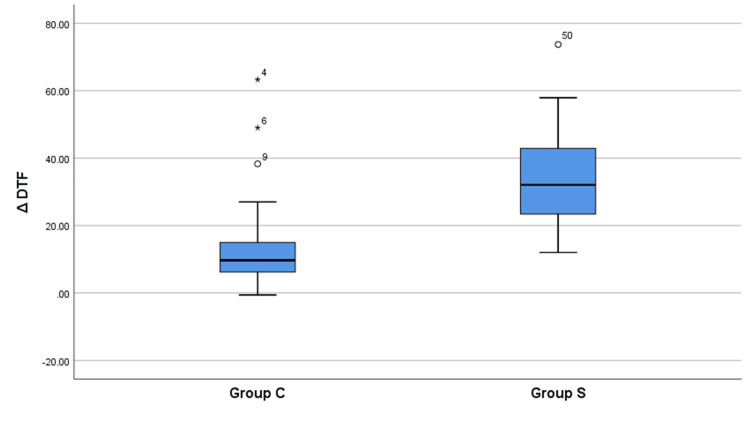
Comparison of the difference in diaphragmatic thickness fraction (ΔDTF, mm) between both groups (n=60) ΔDTF: Change in diaphragmatic thickness fraction; Group C: Costoclavicular block; Group S: Supraclavicular block

Figure 2 compares the change in DTF (ΔDTF) from preblock to postblock between the CCB (Group C) and SCB (Group S) groups. In Group C, the mean ΔDTF was 14.01%, which means the DTF reduced by 14.01% postblock compared to preblock values. In comparison, the mean ΔDTF in Group S was 34.38%, indicating a larger drop of 34.38% in the DTF postblock. The median ΔDTF was 9.7% in Group C and 32.05% in Group S. The Mann-Whitney U test showed the difference in ΔDTF between the two groups was statistically highly significant (p<0.01). To sum up, this figure clearly demonstrates that the SCB led to a significantly higher decline in DTF compared to the CCB. This indicates greater diaphragmatic dysfunction with the supraclavicular approach.

In the costoclavicular group (Group C), the median FVC reduced significantly from 83% predicted preblock to 76% postblock (p<0.01). The median FEV1 declined from 88.5% to 82% (p<0.01). The median PEFR also decreased significantly from 88.5% to 80% after the block (p<0.01). Similarly, in the supraclavicular group (Group S), there were significant reductions in median FVC (88.5%-66%), FEV1 (91%-74%), and PEFR (99%-73%) postblock (p<0.01 for all). However, the declines in PFT parameters were larger in magnitude in the supraclavicular group compared to the costoclavicular group. In a nutshell, pulmonary functions deteriorated significantly in both groups after the nerve blocks. However, the degree of deterioration was more pronounced with the supraclavicular approach indicating greater respiratory dysfunction (Table 5).

**Table 5:** Analysis of the preblock and postblock pulmonary function tests between both groups (n=60)

Pulmonary Function Test	Group	Pulmonary Function Test Value								Test Statistics Wilcoxon Signed-Rank Test	
		Preblock				Postblock					
		Mean	SD	Median	IQR	Mean	SD	Median	IQR	Z value	p value
FVC	Group C	82	7.24	83	13	74.9	8.47	76	9	-3.86	<0.01
	Group S	87.63	10.14	88.5	16	63.17	8.63	66	12	-4.78	<0.01
FEV1	Group C	87.57	11.55	88.5	16	79.63	17.5	82	21	-3.46	<0.01
	Group S	91.23	13.74	91	22	71.2	9.15	74	17	-4.78	<0.01
PEFR	Group C	92.23	14.55	88.5	21	79.3	13.58	80	20	-4.26	<0.01
	Group S	97.97	12.66	99	23	72.97	10.02	73	13	-4.7	<0.01

**Figure 3:**
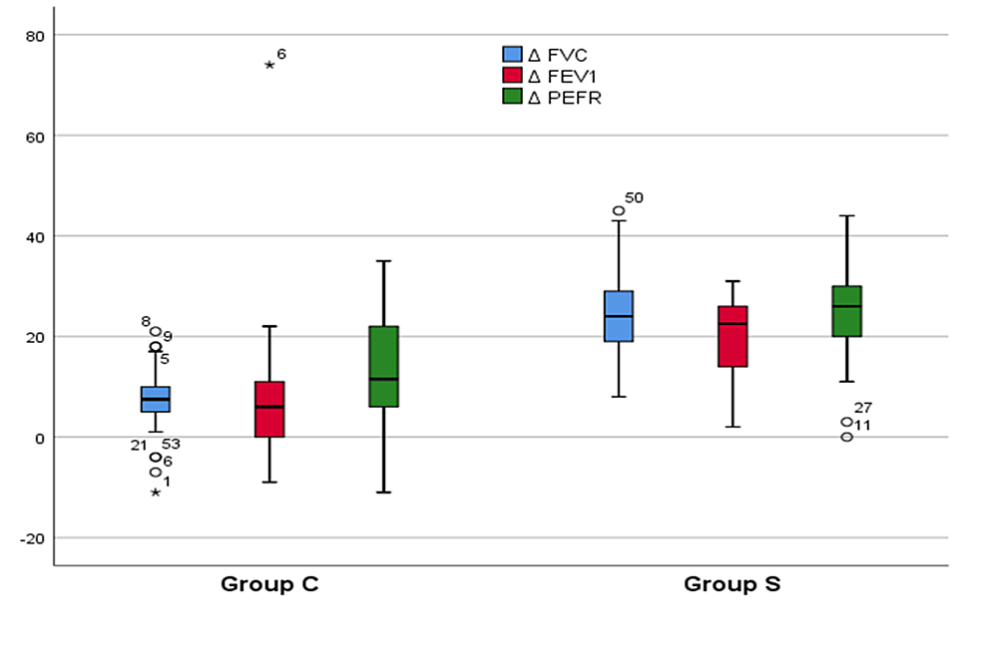
Comparison of the difference in pulmonary function between both groups (n=60) ΔFVC: Change in forced vital capacity; ΔFEV1: Change in forced expiratory volume in 1 second; ΔPEFR: Change in peak expiratory flow ratio; Group C: Costoclavicular block; Group S: Supraclavicular block

Figure 3 compares the change (Δ) in PFT parameters of FVC, FEV1, and PEFR from preblock to postblock between the CCB (Group C) and SCB (Group S) groups. The median ΔFVC was 7.5% in Group C compared to 24% in Group S. This difference was statistically highly significant (p<0.01). Similarly, the median ΔFEV1 was 6% in Group C versus 22.5% in Group S (p<0.01). For ΔPEFR, the median values were 11.5% and 26% in Groups C and S, respectively (p<0.01). In all PFT parameters, the supraclavicular group showed significantly larger declines from preblock to postblock compared to the costoclavicular group. Summarizing these findings, the supraclavicular approach led to greater deterioration of pulmonary functions compared to the costoclavicular approach. This indicates significant respiratory muscle dysfunction with the SCB.

Table 6 compares various block characteristics such as onset time, duration, and time to first analgesia demand between the CCB (Group C) and SCB (Group S) groups. The mean sensory block onset time was 13.18 minutes in Group C and 11.73 minutes in Group S. The difference was not statistically significant (p=0.31). The median motor block onset time was 27 minutes in Group C and 26 minutes in Group S. No significant difference was observed (p=0.68).

**Table 6:** Comparison of block characteristics between both groups (n=60) SD: Standard deviation; IQR: Interquartile range

Block Characteristics	Time (Minutes)								Test Statistics Independent t-Test	
	Group C (Costoclavicular block) [n=30]				Group S (Supraclavicular block) [n=30]					
	Mean	SD	Median	IQR	Mean	SD	Median	IQR	t value	p value
Sensory block onset	13.18	5.64	13.5	7.5	11.73	5.5	11.5	7.3	1.01	0.31
Motor block onset	27.48	7.72	27	13.1	26.56	9.69	26	11	0.41	0.68
Duration of sensory block	424.43	87.45	420	137	445.97	103.22	434	131	-0.87	0.38
Duration of motor block	507.13	107.73	496.5	128	585.33	155.53	573	211	Z=1.93*	0.053
Time to first demand of analgesia	432.4	91.2	429	157	460.33	107.43	441	176	-1.08	0.28
*Mann Whitney U test was applied as the duration of motor block data did not follow a normal distribution.										

The median duration of sensory block was 420 minutes in Group C versus 434 minutes in Group S. The difference was not significant statistically (p=0.38). The median duration of the motor block was longer in Group S (573 minutes) compared to Group C (496.5 minutes). However, this did not reach statistical significance (p=0.053).

The time to first analgesic demand had median values of 429 minutes and 441 minutes in Groups C and S, respectively. The difference was not significant (p=0.28).

**Figure 4:**
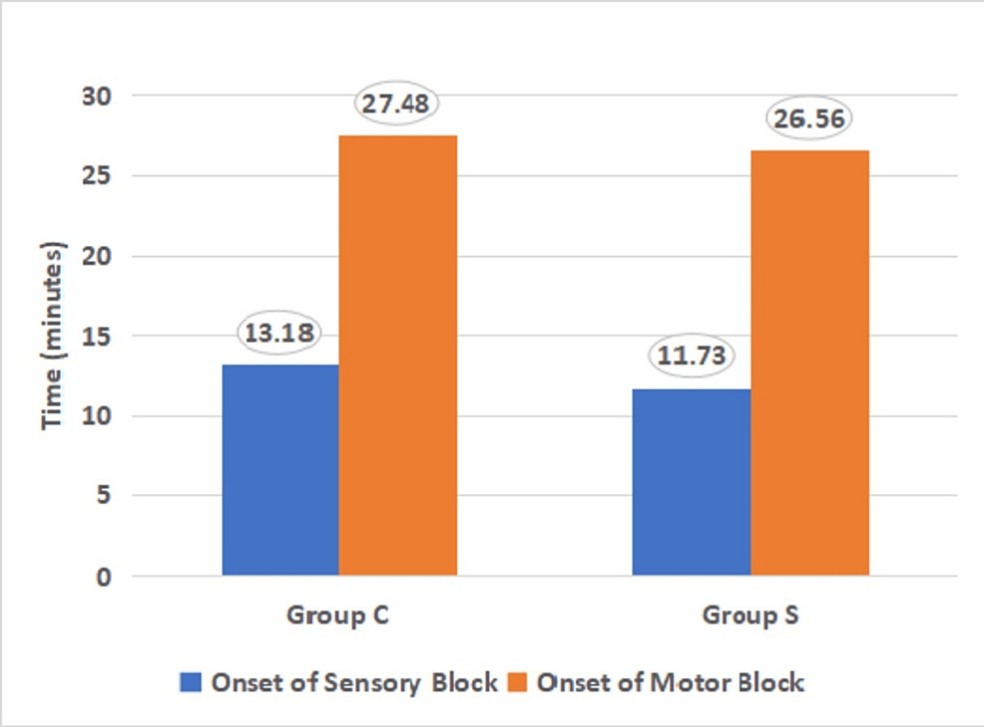
Comparison of the mean time for the onset of sensory and motor blocks between both groups (n=60) Group C: Costoclavicular block; Group S: Supraclavicular block

Figure 4 shows a comparison of the mean time for the onset of the sensory and motor blocks between both groups.

**Figure 5:**
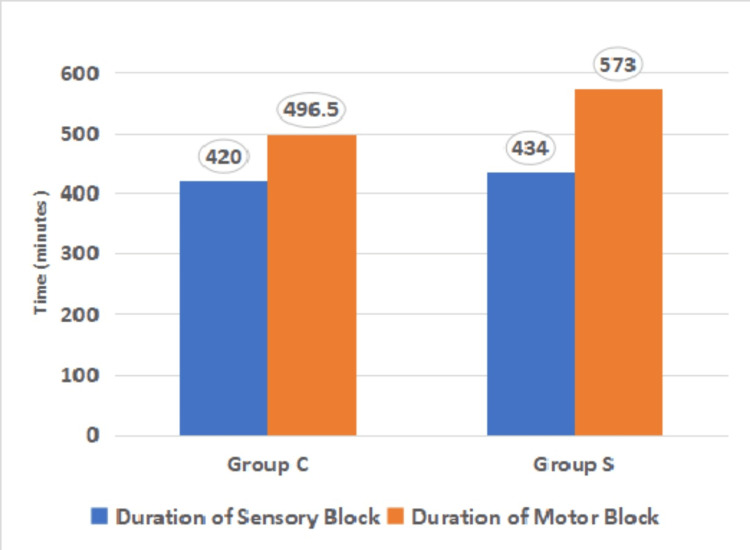
Comparison of the median time for the duration of the sensory and motor blocks between both groups (n=60) Group C: Costoclavicular group; Group S: Supraclavicular group

Figure 5 shows a comparison of the median time for the duration of the sensory and motor blocks between both groups.

To put these findings concisely, there were no statistically significant differences between the two approaches regarding block onset, duration, or analgesic requirements. The block characteristics were comparable between the costoclavicular and supraclavicular approaches.

## Discussion

The present prospective, randomized, observer-blinded study comprehensively evaluated the impact of ultrasound-guided supraclavicular and costoclavicular brachial plexus blocks on diaphragmatic function and pulmonary parameters in patients undergoing below-shoulder surgeries. The findings revealed superior preservation of diaphragmatic contractility and lesser deterioration of PFTs with the costoclavicular approach compared to the SCB.

Kaufman et al. reported a case series of patients requiring surgical treatment for permanent diaphragm paralysis after inter scalene nerve block for shoulder surgery, highlighting the severe consequences of phrenic nerve involvement [11].

The mechanism behind diaphragmatic dysfunction following SCB involves the spread of LA to the phrenic nerve, which arises from the C3-C5 nerve roots and runs in close proximity to the trunks of the brachial plexus [12]. In contrast, the costoclavicular approach targets the cords of the brachial plexus at a level below the phrenic nerve, thereby minimizing the risk of inadvertent phrenic nerve blockade [13]. 

The diaphragmatic function was meticulously assessed through ultrasonographic evaluation of diaphragm thickness during inspiration and expiration, pre- and postblock. A decline in DTF signifies impaired diaphragmatic excursion and contractility. The supraclavicular group exhibited a substantial reduction in DTF from pre-block to post-block (mean ΔDTF: 34.38%), indicating significant diaphragmatic dysfunction. In contrast, the costoclavicular group showed a significantly less decline in DTF (mean ΔDTF: 14.01%), suggesting better preservation of diaphragmatic function. These findings corroborate several previous studies that have reported a higher incidence of HDP following supraclavicular blocks due to potential phrenic nerve involvement [14-16].

These findings align with a study by Tran et al. [14], which demonstrated a significantly lower incidence of HDP with the costoclavicular approach (0%) compared to the supraclavicular technique (67%).

While diaphragmatic dysfunction can occur with both techniques, the present study and previous literature suggest a lower risk with the costoclavicular approach [14,17]. However, it is noteworthy that the CCB may be technically more challenging, particularly in patients with anatomical variations or challenging ultrasound visualization [13,17].

PFTs were assessed to evaluate the impact of diaphragmatic dysfunction on respiratory mechanics. Both groups exhibited significant declines in FVC, FEV1, and PEFR following the nerve blocks. However, the magnitude of deterioration was significantly greater in the supraclavicular group compared to the costoclavicular group. These findings are consistent with several previous studies that have reported reductions in PFT parameters, particularly FVC, following brachial plexus blocks, attributable to diaphragmatic paralysis [18-21].

Urmey et al. reported a 100% incidence of HDP associated with interscalene brachial plexus anesthesia, as diagnosed by ultrasonography, leading to significant reductions in FVC and FEV1 [18]. Also observed were substantial decreases in FVC, FEV1, and PEFR following interscalene brachial plexus block for shoulder surgery, with larger reductions associated with higher volumes of LA [21]. These findings underscore the impact of diaphragmatic dysfunction on pulmonary mechanics and highlight the importance of minimizing phrenic nerve involvement during brachial plexus blocks.

The impaired diaphragmatic excursion and contractility following brachial plexus blocks, particularly with the supraclavicular approach, can be attributed to the potential involvement of the phrenic nerve due to its proximity to the SCB. The resultant HDP leads to a restrictive pulmonary defect, manifesting as reduced lung volumes and diminished airflow rates. This phenomenon is particularly concerning in patients with pre-existing respiratory compromise, as the superimposed diaphragmatic dysfunction can precipitate respiratory failure and potentially life-threatening complications [9,22].

The volume of LA used during brachial plexus blocks is another factor that can influence the extent of diaphragmatic involvement and respiratory impairment. Urmey et al. concluded that reducing the volume of LA agent used significantly lessens the compromise in pulmonary function [22].

Characteristics such as onset and duration of the block are also important considerations in the choice of the brachial plexus block technique. Previous studies have reported variable findings regarding the onset and duration of blocks with different approaches [23].

Zhang et al. concluded that proportions of the complete sensory and motor blocks at each interval after injection showed no significant difference when the groups were compared, which is consistent with our study [24]. There was no significant difference regarding the onset and duration of both sensory and motor blocks as well as the time to first demand of analgesia in our study (Table 6).

The findings of this study have important clinical implications, particularly in the context of ambulatory and outpatient surgeries, where patients are discharged soon after the procedure. Diaphragmatic dysfunction and respiratory impairment may not be immediately apparent, potentially leading to delayed recognition and management of associated complications [1]. Therefore, careful selection of the brachial plexus block technique, patient monitoring, and appropriate counselling are crucial to mitigate the risk of adverse respiratory events.

While the costoclavicular approach demonstrated better preservation of diaphragmatic function and pulmonary parameters in this study, it is important to note that the technique is not devoid of potential complications. As previously mentioned, reports of phrenic nerve palsy, albeit rare, have been documented with the CCB. Additionally, the costoclavicular approach may be technically more challenging, particularly in patients with anatomical variations or challenging ultrasound visualization.

While the present study did not investigate the impact of local anesthetic volume, future research could explore the optimal volume for costoclavicular and supraclavicular blocks to minimize diaphragmatic dysfunction and respiratory impairment.

In conclusion, this prospective, randomized, observer-blinded study contributes important evidence to the existing literature on the differential effects of supraclavicular and costoclavicular brachial plexus blocks on diaphragmatic function and pulmonary parameters. The costoclavicular approach demonstrated superior preservation of diaphragmatic contractility and lesser deterioration of PFTs compared to the supraclavicular block. These findings are supported by previous studies and emphasize the importance of careful technique selection, patient monitoring, and counselling to mitigate the risk of respiratory complications associated with brachial plexus blocks, particularly in patients with pre-existing respiratory compromise.

Limitations of the study

The study has several limitations that warrant consideration. Firstly, the relatively small sample size of 60 patients from a single center may limit the generalizability of the findings. Potential confounding factors, such as concomitant medication, which could influence the diaphragmatic function and pulmonary parameters, were not accounted for. The study also did not consider the cost-effectiveness or resource utilization associated with each block technique, which could be an important consideration for clinical decision-making and resource allocation.

## Conclusions

This prospective, randomized, observer-blinded study provides valuable insights into the differential effects of ultrasound-guided supraclavicular and costoclavicular brachial plexus blocks on the diaphragmatic function and pulmonary parameters. The findings demonstrate superior preservation of diaphragmatic contractility and lesser deterioration of PFTs with the costoclavicular approach compared to the SCB. These results align with previous literature highlighting the lower risk of phrenic nerve involvement and subsequent diaphragmatic dysfunction with the costoclavicular technique.

The CCB emerges as an equally effective, yet potentially safer alternative for patients undergoing below-shoulder surgeries, particularly those with pre-existing respiratory compromise or at higher risk for respiratory complications. However, it is essential to consider the technical challenges associated with the costoclavicular approach and the potential, albeit lesser risk of phrenic nerve palsy. Overall, this study contributes to the growing body of evidence supporting the use of diaphragm-sparing regional anaesthesia techniques for surgeries around the shoulder. Careful patient selection, thorough assessment of risk factors, and judicious choice of technique remain paramount in optimizing patient outcomes and minimizing the risk of adverse respiratory events associated with brachial plexus blocks.
